# Advanced age is not the decisive factor in chemotherapy of small cell lung cancer: a population-based study

**DOI:** 10.18632/aging.204114

**Published:** 2022-06-08

**Authors:** Hanyu Rao, Shunping Zhou, Aihong Mei, Anjie Yao, Shuanshuan Xie

**Affiliations:** 1 Department of Respiratory Medicine, Shanghai 10th People’s Hospital, Tongji University School of Medicine, Shanghai 200072, China; 2 Tongji University School of Medicine, Shanghai 200092, China; 3 Department of Cardiology Medicine, Yangpu Hospital, Tongji University School of Medicine, Shanghai 200090, China

**Keywords:** small cell lung cancer (SCLC), Surveillance, Epidemiology and End Results (SEER), chemotherapy, overall survival, lung-cancer specific survival

## Abstract

Objective: There is limited research on the impact of chemotherapy on the prognosis of different age group patients with small cell lung cancer (SCLC). The aim of this study was to explore the impact of chemotherapy on survival prognosis of elderly patients with SCLC.

Methods: Based on the Surveillance, Epidemiology and End Results (SEER) database, 57,460 SCLC patients between 2004 and 2015 were identified and divided into a ≤ 80 years group (*n* = 50,941) and a >80 years group (*n* = 6,519). Confounding factors were controlled by propensity score matching (PSM) analysis. Kaplan Meier (KM) analysis was performed to determine the impact of chemotherapy on overall survival (OS) and lung-cancer specific survival (LCSS) of the patients. Other variables that could affect survival of SCLC patients were also examined by COX analysis.

Results: KM analysis showed that both OS and LCSS were improved in chemotherapy group compared to those in non-chemotherapy group (log rank *P* < 0.001) in both age groups after PSM. Cox analysis demonstrated the survival benefit of chemotherapy in both ≤ 80 years group (OS: HR 0.435; 95% CI 0.424–0.447; LCSS: HR 0.436; 95% CI 0.424–0.448) and >80 years group (OS: HR 0.424; 95% CI 0.397–0.451; LCSS: HR 0.415; 95% CI 0.389–0.444). Additionally, the following parameters had a negative impact on survival of elderly patients: male sex, tumor location in main bronchus, increased stage, bilateral tumor, no surgery or radiation, and lower median household income.

Conclusions: Elderly patients with SCLC should be encouraged to receive chemotherapy provided their general conditions permit.

## INTRODUCTION

According to the American Cancer Society (ACS), lung cancer is the second most common cancer, with 235,760 new cases diagnosed in 2021 [1]. It is also one of the cancers with the lowest 5-year relative survival rate (21%), especially for small cell lung cancer (SCLC) whose 2-year survival is even lower than that of non-small cell lung cancer (NSCLC) (14–15% vs. 42%) [1]. SCLC accounts for approximately 13% of all lung carcinomas [2]. Compared with NSSLC, SCLC is associated with poorer prognosis, earlier and more frequent brain metastasis [3, 4]. Lung cancer is also a cancer of the elderly, with a median age of about 71 years at diagnosis [5], and there is an increasing trend in the proportion of SCLC patients in the age group older than 75 years according to a study from Hebei, China [6].

Over the past three decades, chemotherapy has provided considerable survival benefits for SCLC patients [7], and remains the standard treatment for first- and second-line management of SCLC [8]. Immunotherapy also plays an important role in patients with relapsed SCLC [8]. There have been many clinical trials on immune checkpoints inhibitors (ICIs), tumor vaccines, antigenic targets, and adoptive cellular immunotherapy in SCLC, but the results have been somewhat disappointing so far [9]. Treatment planning and decision mainly depend on the cancer TNM staging system and the Veterans Administration Lung Study Group (VALG) staging system, according to the National Comprehensive Cancer Network Clinical Practice Guidelines for SCLC, the Chinese Society of Clinical Oncology Lung Cancer Guidelines, and the European Society for Medical Oncology Clinical Practice Guidelines for Metastatic SCLC [10]. With the overall population life expectancy improving, treatment for elderly patients has aroused increasing attention. Given the performance status (PS) of the elderly and the associated comorbidities and toxicity, some oncologists are not inclined to using chemotherapy in elderly patients with SCLC [11]. So, whether chemotherapy is beneficial to elderly patients needs to be further confirmed. Several retrospective cohort studies exploring the relationship between age and treatment choice have defined elderly patients as age ≥ 65 [12, 13], ≥ 70 [14, 15] or ≥ 75 years old [11, 16]. There is limited research on patients > 80 years old, much less in elderly SCLC patients older than 80 years. A recent single center retrospective study evaluated the survival outcome associated with the treatment strategies in 56 cancer patients aged over 80 years, but only 7 of them were SCLC patients [17]. The aim of present study is to explore the impact of chemotherapy on survival prognosis of elderly patients with SCLC using the Surveillance, Epidemiology and End Results (SEER) database.

## METHODS

### Data source

We performed this study to verify the relationship between chemotherapy and the survival prognosis of SCLC patients of different ages. All the data were based on the Surveillance, Epidemiology, and End Results (SEER) database, which was established in 1973 and collects information on cancer incidences and survival in the United States (US), covering 17 population-based cancer registries, accounting for about 28% of the current US population [18].

### Study population

**Figure 1:**
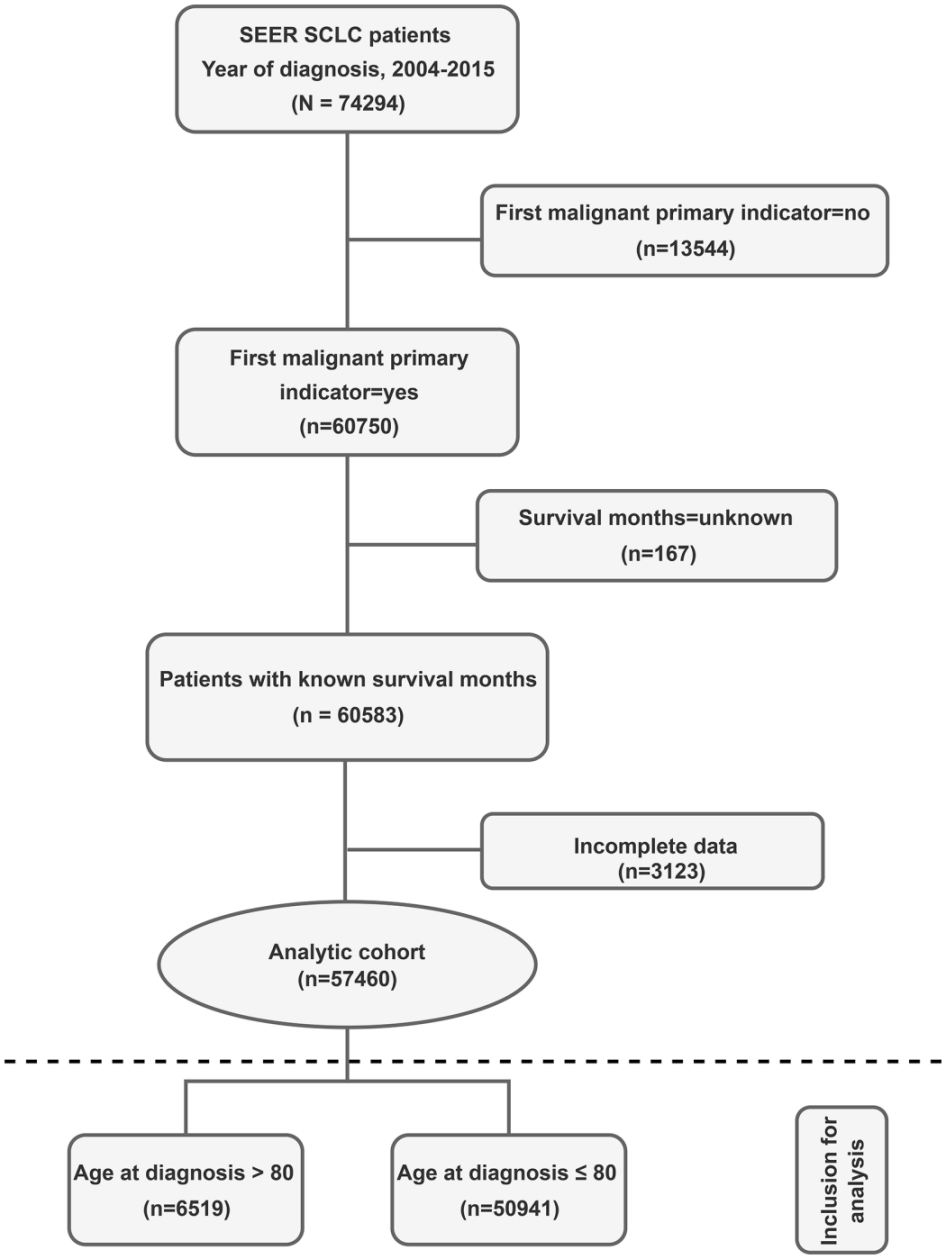
**Study flow diagram.** Abbreviations: SCLC: Small cell lung cancer; SEER: Surveillance, Epidemiology, and End Results registry.

From 74,294 SCLC patients initially identified from the SEER database between 2004 and 2015, we included 57,460 patients for final analysis after excluding 13,544 patients without first malignant primary indicators, 167 with unknown survival months and 3,123 with incomplete data according to the inclusion and exclusion criteria (Figure 1). They were divided into two age groups: ≤ 80 years group (*n* = 50,941) and >80 years group (*n* = 6,519), and each group was further divided into chemotherapy group (*n* = 37,136 for ≤ 80 and 2,774 for >80 years) and non-chemotherapy group (*n* = 13,805 for ≤ 80 and 3,745 for > 80 years).

### Covariates

Baseline clinical characteristics including median age at diagnosis, gender, race, region, year of diagnosis, primary site, grade, laterality, stage, radiation, surgery, marital status, education level and median household income were collected.

### Statistical analyses

Continuous variables were compared using *t*-test, and categorical variables were compared using chi-square. Potential deviation between chemotherapy and non-chemotherapy groups was controlled by propensity score matching (PSM) analysis. Kaplan-Meier (KM) analysis and the log rank test were applied to compare overall survival (OS) and lung-cancer specific survival (LCSS) between patients with or without chemotherapy. To study whether other variables could affect survival of SCLC patients, COX analysis was performed in each group. Statistical significance was set at a two-tailed *P* value < 0.05. All analyses were performed with IBM SPSS version 25.0.

## RESULTS

### Study cohort characteristics

**Figure 2:**
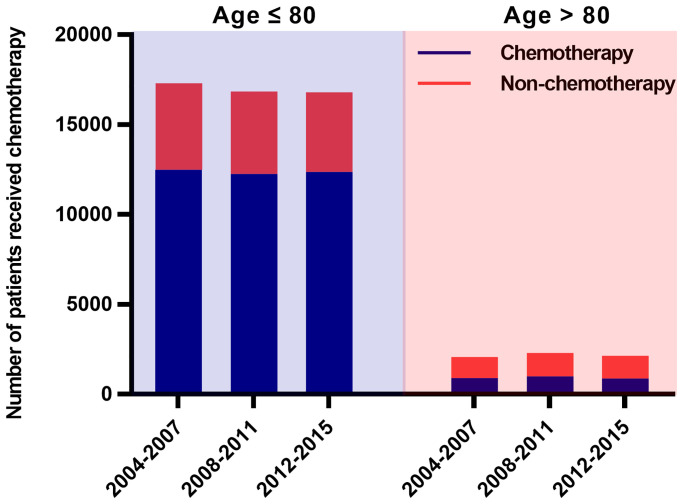
**Numbers of SCLC patients who received chemotherapy over time (2004–2015).** There were 12,499, 12,264 and 12,373 SCLC patients aged ≤ 80 years who received chemotherapy in 2004–2007, 2008–2011 and 2012–2015, accounting for 72%, 73% and 74% of all young patients respectively. During the same periods, 907 (44%), 991 (43%) and 876 (41%) patients older than 80 years underwent chemotherapy respectively. The proportion of patients receiving chemotherapy did not change significantly over time. Abbreviations: SCLC: Small cell lung cancer.

Demographic and clinical characteristics of the patients are included in Table 1. Among these, 50,941 were ≤ 80 years old and 6,519 were >80 years old, and their median age at diagnosis was 65 years (interquartile range, 59–72 years) and 83 years (interquartile range, 81–85 years) respectively. Both groups of patients were distributed roughly equally by gender (men 50.4%, women 49.6% in ≤ 80 years group; men 47.0%, women 53.0% in >80 years group). Most patients were Caucasian (86.9% in ≤80 years group; 87.8% in >80 years group), and from the East (50.8% in ≤80 years group; 41.6% in >80 years group) or Southwest (36.5% in ≤80 years group; 45.1% in >80 years group), only a few (0.2% in ≤80 years group; 0.1% in >80 years group) were living in the Northwest. Most patients did not receive surgical treatment (96.8% in ≤80 years group; 98.0% in >80 years group) or radiotherapy (51.1% in ≤80 years group; 74.4% in >80 years group). Patients in the early stage, married, treated with radiation but not surgery and had better education were more likely to receive chemotherapy. About 43% of the elderly patients in our study received chemotherapy. Most (73%) of the younger patients chose chemotherapy (Table 1). The proportion of SCLC patients choosing chemotherapy did not vary significantly with the year of diagnosis (Figure 2).

**Table 1:** Demographic and clinical characteristics of patients with SCLC.

**Variable**	**Age groups**							
	**Age ≤ 80 year Chemotherapy**				**Age > 80 year Chemotherapy**			
	**Yes *n* = 37136 (73)**	**No *n* = 13805 (27)**	**Total *n* = 50941 (100)**	***P***	**Yes *n* = 2774 (43)**	**No *n* = 3745 (57)**	**Total *n* = 6519 (100)**	***P***
Median age at diagnosis	64 (58–71)	68 (61–74)	65 (59–72)	<0.001	82 (81–84)	83 (81–86)	83 (81–85)	<0.001
Gender								
Male	18649 (50.2)	7002 (50.7)	25651 (50.4)	0.314	1381 (49.8)	1686 (45.0)	3067 (47.0)	<0.001
Female	18487 (49.8)	6803 (49.3)	25290 (49.6)		1393 (50.2)	2059 (55.0)	3452 (53.0)	
Race recode								
White	32288 (86.9)	11972 (86.7)	44260 (86.9)	0.017	2447 (88.2)	3275 (87.4)	5722 (87.8)	0.097
Black	3376 (9.1)	1331 (9.6)	4707 (9.2)		156 (5.6)	259 (6.9)	415 (6.4)	
Other	1441 (3.9)	483 (3.5)	1924 (3.8)		170 (6.1)	207 (5.5)	377 (5.8)	
Unknown	31 (0.1)	19 (0.1)	50 (0.1)		1 (0.0)	4 (0.1)	5 (0.1)	
CHSDA region								
EAST	19175 (51.6)	6678 (48.4)	25853 (50.8)	<0.001	1210 (43.6)	1501 (40.1)	2711 (41.6)	<0.001
NORTHWEST	81 (0.2)	15 (0.1)	96 (0.2)		3 (0.1)	2 (0.1)	5 (0.1)	
SOUTHWEST	12904 (34.7)	5704 (41.3)	18608 (36.5)		1162 (41.9)	1779 (47.5)	2941 (45.1)	
NORTH	4976 (13.4)	1408 (10.2)	6384(12.5)		399 (14.4)	463 (12.4)	862 (13.2)	
Year of diagnosis								
2004–2007	12499 (33.7)	4796 (34.7)	17295 (34.0)	0.013	907 (32.7)	1164 (31.1)	2071 (31.8)	0.097
2008–2011	12264 (33.0)	4587 (33.2)	16851 (33.1)		991 (35.7)	1305 (34.8)	2296 (35.2)	
2012–2017	12373 (33.3)	4422 (32.0)	16795 (33.0)		876 (31.6)	1276 (34.1)	2152 (33.0)	
Primary Site								
Upper lobe	17512 (47.2)	5866 (42.5)	23378 (45.9)	<0.001	1188 (42.8)	1403 (37.5)	2591 (39.7)	<0.001
Middle lobe	1431 (3.9)	514 (3.7)	1945 (3.8)		118 (4.3)	159 (4.2)	277 (4.2)	
Lower lobe	6941 (18.7)	2522 (18.3)	9463 (18.6)		690 (24.9)	851 (22.7)	1541 (23.6)	
NOS	6301 (17.0)	3173 (23.0)	9474 (18.6)		482 (17.4)	939 (25.1)	1421 (21.8)	
Overlapping lesion	559 (1.5)	226 (1.6)	785 (1.5)		54 (1.9)	51 (1.4)	105 (1.6)	
Main bronchus	4392 (11.8)	1504 (10.9)	5896 (11.6)		242 (8.7)	342 (9.1)	584 (9.0)	
Grade								
I	53 (0.1)	29 (0.2)	82 (0.2)	0.019	0 (0.0)	7 (0.2)	7 (0.1)	0.027
II	116 (0.3)	57 (0.4)	173 (0.3)		6 (0.2)	12 (0.3)	18 (0.3)	
III	3239 (8.7)	1224 (8.9)	4463 (8.8)		221 (8.0)	303 (8.1)	524 (8.0)	
IV	6948 (18.7)	2449 (17.7)	9397 (18.4)		556 (20.0)	666 (17.8)	1222 (18.7)	
Unknown	26780 (72.1)	10046 (72.8)	36826 (72.3)		1991 (71.8)	2757 (73.6)	4748 (72.8)	
Laterality								
Right-origin of primary	20451 (55.1)	7137 (51.7)	27588 (54.2)	<0.001	1528 (55.1)	1996 (53.3)	3524 (54.1)	<0.001
Left-origin of primary	14731 (39.7)	5385 (39.0)	20116 (39.5)		1096 (39.5)	1407 (37.6)	2503 (38.4)	
Paired sit	1375 (3.7)	931 (6.7)	2306 (4.5)		104 (3.7)	253 (6.8)	357 (5.5)	
Only one side - side unspecified	161 (0.4)	101 (0.7)	262 (0.5)		8 (0.3)	27 (0.7)	35 (0.5)	
Not a paired site	79 (0.2)	29 (0.2)	108 (0.2)		8 (0.3)	8 (0.2)	16 (0.2)	
Bilateral single primary	339 (0.9)	222 (1.6)	561 (1.1)		30 (1.1)	54 (1.4)	84 (1.3)	
Stage Group								
I	1416 (3.8)	597 (4.3)	2013 (4.0)	<0.001	156 (5.6)	207 (5.5)	363 (5.6)	<0.001
II	842 (2.3)	182 (1.3)	1024 (2.0)		61 (2.2)	56 (1.5)	117 (1.8)	
III	11795 (31.8)	2880 (20.9)	14675 (28.8)		884 (31.9)	1022 (27.3)	1906 (29.2)	
IV	23083 (62.2)	10146 (73.5)	33229 (65.2)		1673 (60.3)	2460 (65.7)	4133 (63.4)	
Radiation recode								
Yes	22007 (59.3)	2890 (20.9)	24897 (48.9)	<0.001	1073 (38.7)	594 (15.9)	1667 (25.6)	<0.001
No	15129 (40.7)	10915 (79.1)	26044 (51.1)		1701 (61.3)	3151 (84.1)	4852 (74.4)	
Surgery								
Yes	1117 (3.0)	512 (3.7)	1629 (3.2)	<0.001	39 (1.4)	94 (2.5)	133 (2.0)	0.002
No	36019 (97.0)	13293 (96.3)	49312 (96.8)		2735 (98.6)	3651 (97.5)	6386 (98.0)	
Marital status								
Married	19567 (52.7)	6015 (43.6)	25582 (50.2)	<0.001	1257 (45.3)	1356 (36.2)	2613 (40.1)	<0.001
Single	5024 (13.5)	2386 (17.3)	7410 (14.5)		162 (5.8)	255 (6.8)	417 (6.4)	
Divorced	5777 (15.6)	2060 (14.9)	7837 (15.4)		186 (6.7)	258 (6.9)	444 (6.8)	
Windowed	5001 (13.5)	2505 (18.1)	7506 (14.7)		1073 (38.7)	1689 (45.1)	2762 (42.4)	
Unknown	1211 (3.3)	630 (4.6)	1841 (3.6)		86 (3.1)	166 (4.4)	252 (3.9)	
Unmarried or domestic partner	65 (0.2)	16 (0.1)	81 (0.2)		0 (0.0)	2 (0.1)	2 (0.0)	
Separated	491 (1.3)	193 (1.4)	684 (1.3)		10 (0.4)	19 (0.5)	29 (0.4)	
High school education								
>21	6990 (18.8)	3338 (24.2)	10328 (20.3)	<0.001	445 (16.0)	730 (19.5)	1175 (18.0)	<0.001
13–20	11319 (30.5)	4366 (31.6)	15685 (30.8)		790 (28.5)	1103 (29.5)	1893 (29.0)	
7–12	16421 (44.2)	5385 (39.0)	21806 (42.8)		1320 (47.6)	1611 (43.0)	2931 (45.0)	
≤7	2406 (6.5)	716 (5.2)	3122 (6.1)		219 (7.9)	301 (8.0)	520 (8.0)	
Median household income								
≤38000	3626 (9.8)	1440 (10.4)	5066 (9.9)	0.001	126 (4.5)	208 (5.6)	334 (5.1)	0.051
38000–47999	7513 (20.2)	2792 (20.2)	10305 (20.2)		452 (16.3)	590 (15.8)	1042 (16.0)	
48000–62999	14408 (38.8)	5489 (39.8)	19897 (39.1)		1062 (38.3)	1511 (40.3)	2573 (39.5)	
>63000	11589 (31.2)	4084 (29.6)	15673 (30.8)		1134 (40.9)	1436 (38.3)	2570 (39.4)	

Abbreviations: SCLC: Small cell lung cancer; NOS: Not otherwise specified.

### Comparison of survival curves between chemotherapy group and non-chemotherapy group

**Figure 3:**
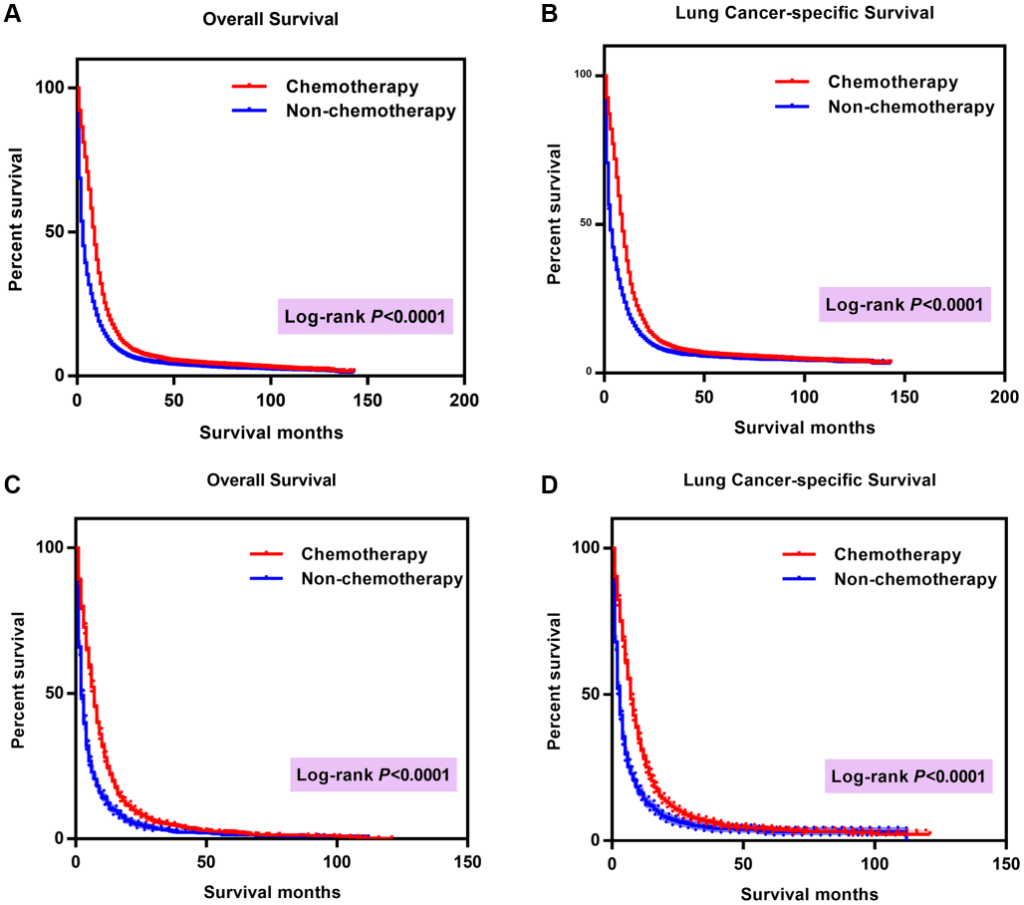
**Comparison of survival curves between chemotherapy group and group without chemotherapy.** (**A**) Comparison of OS in patients ≤ 80 years old; (**B**) Comparison of LCSS in patients ≤ 80 years old; (**C**) Comparison of OS in patients >80 years old; (**D**) Comparison of LCSS in patients > 80 years old. Abbreviations: OS: Overall survival; LCSS: Lung cancer-specific survival; Cum: Cumulative.

Enrolled in the study were 27,486 patients ≤ 80 years old and 4,550 patients >80 years old after propensity score matched analysis. KM analysis demonstrated improved OS and LCSS in patients of chemotherapy group compared to patients of non-chemotherapy group, with the survival curves showing statistically significant differences (log rank *P* < 0.0001) in both ≤ 80 years group and > 80 years group (Figure 3).

### The effects of chemotherapy on the survival of patients with SCLC

**Figure 4:**
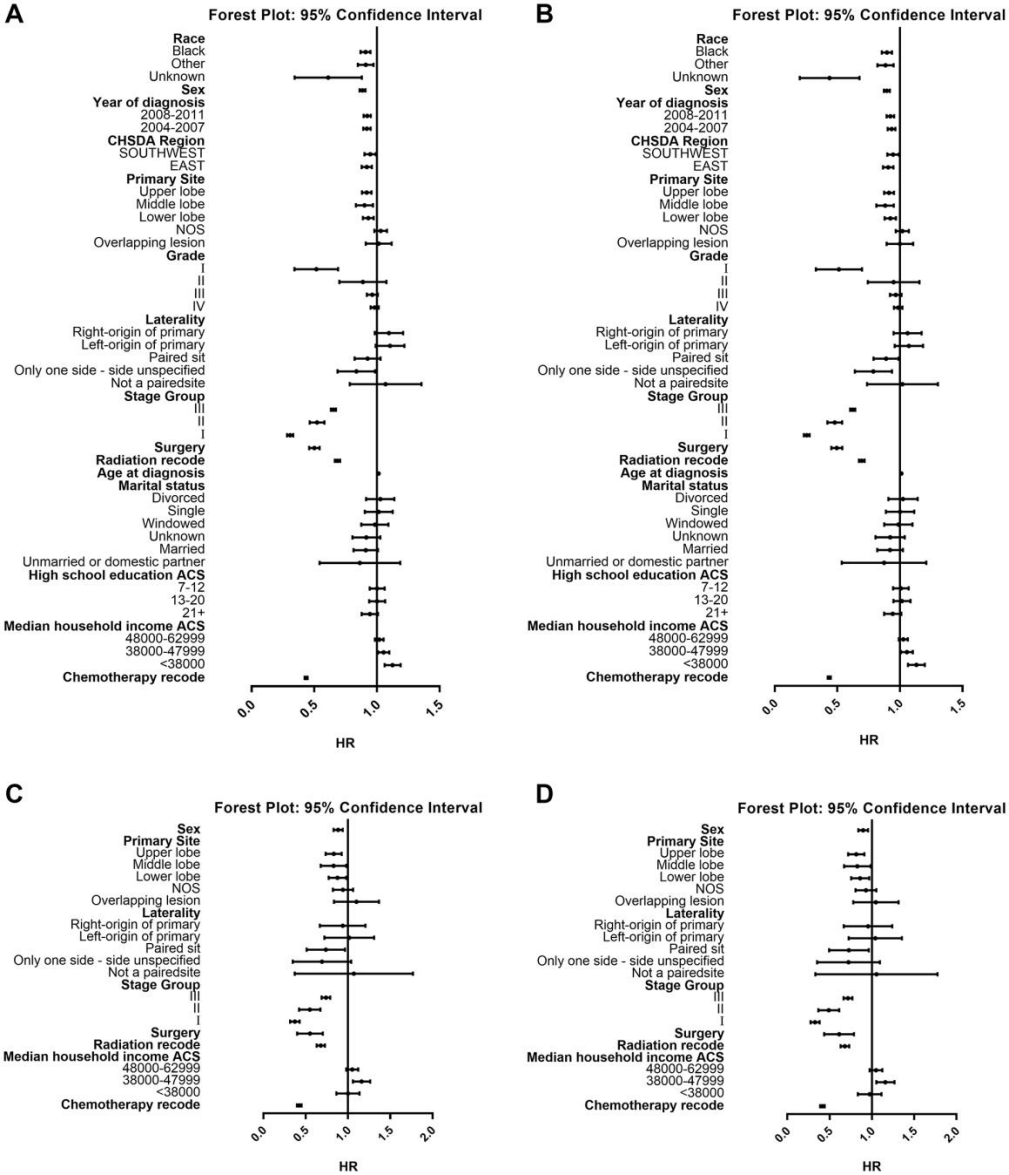
**Forest plot of HRs of factors that can influence OS or LCSS in patients ≤ or >80.** (**A**) HRs of factors influencing OS in patients ≤80 years old; (**B**) HRs of factors influencing LCSS in patients ≤80 years old; (**C**) HRs of factors influencing OS in patients >80 years old; (**D**) HRs of factors influencing LCSS in patients >80 years old. Abbreviations: HRs: Hazard ratios; OS: Overall survival; LCSS: Lung cancer-specific survival.

Cox analysis demonstrated the survival benefit of chemotherapy in both ≤ 80 years group (OS: HR 0.435; 95% CI 0.424–0.447; *P* < 0.001; LCSS: HR 0.436; 95% CI 0.424–0.448; *P* < 0.001) and > 80 years group (OS: HR 0.424; 95% CI 0.397–0.451; *P* < 0.001; CSS: HR 0.415; 95% CI 0.389–0.444; *P* < 0.001). Also, survival varied with the change of other variables including sex, primary site, laterality, stage, surgery, radiation and household income. Additionally, variables like race, year of diagnosis, region, age, marital status, and education status also affected survival in in ≤ 80 years group (Tables 2 and 3; Figure 4). The following parameters had a negative impact on survival of patients in ≤ 80 years group: Caucasian, male sex, later year of diagnosis, older age, north region, tumor location in main bronchus, increased stage and grade, bilateral tumor, no surgery or radiation, separation, lower median household income and poorer educated, while the following parameters had a negative impact on survival of patients in > 80 years group: male sex, tumor location in main bronchus, increased stage, bilateral tumor, no surgery or radiation, and lower median household income.

**Table 2:** Multivariate analysis using a Cox proportional hazards model in SCLC patients ≤ 80.

**Variable**	**Multivariate analysis**					
	**OS**			**CSS**		
	**HR**	**95% CI**	***P***	**HR**	**95% CI**	***P***
Race recode			<0.001			<0.001
White	Reference					
Black	0.909	0.870 to 0.949	<0.001	0.896	0.856 to 0.938	<0.001
Other	0.909	0.849 to 0.974	0.007	0.884	0.822 to 0.951	0.001
Unknown	0.572	0.365 to 0.898	0.015	0.395	0.224 to 0.696	0.001
Sex			<0.001			
Male	Reference					
Female	0.885	0.863 to 0.908	<0.001	0.896	0.872 to 0.920	<0.001
Year of diagnosis			<0.001			<0.001
2012-2015	Reference					
2008-2011	0.922	0.894 to 0.951	<0.001	0.924	0.894 to 0.955	<0.001
2004-2007	0.920	0.891 to 0.950	<0.001	0.933	0.903 to 0.965	<0.001
CHSDA Region			0.001			<0.001
NORTH	Reference					
SOUTHWEST	0.946	0.902 to 0.992	0.023	0.946	0.900 to 0.993	<0.001
EAST	0.919	0.879 to 0.960	<0.001	0.907	0.866 to 0.949	<0.001
Primary Site			<0.001			<0.001
Main bronchus	Reference					
Upper lobe	0.918	0.881 to 0.957	<0.001	0.913	0.874 to 0.953	<0.001
Middle lobe	0.899	0.834 to 0.969	0.005	0.880	0.814 to 0.952	0.001
Lower lobe	0.931	0.888 to 0.975	0.003	0.923	0.879 to 0.969	0.001
NOS	1.029	0.980 to 1.081	0.253	1.019	0.968 to 1.072	0.476
Overlapping lesion	1.011	0.913 to 1.120	0.836	0.996	0.896 to 1.108	0.941
Grade			<0.001			<0.001
Unknown	Reference					
I	0.497	0.353 to 0.700	<0.001	0.491	0.341 to 0.708	<0.001
II	0.876	0.708 to 1.083	0.222	0.935	0.752 to 1.163	0.545
III	0.963	0.920 to 1.007	0.101	0.966	0.922 to 1.013	0.156
IV	0.982	0.950 to 1.014	0.265	0.986	0.953 to 1.020	0.43
Laterality			<0.001			<0.001
Bilateral, single primary	Reference					
Right-origin of primary	1.092	0.985 to 1.211	0.096	1.058	0.952 to 1.175	0.297
Left-origin of primary	1.101	0.993 to 1.222	0.069	1.068	0.960 to 1.187	0.224
Paired sit	0.922	0.825 to 1.030	0.152	0.886	0.791 to 0.992	0.036
Only one side - side unspecified	0.827	0.691 to 0.991	0.04	0.779	0.645 to 0.941	0.010
Not a paired site	1.044	0.797 to 1.368	0.755	0.995	0.751 to 1.317	0.970
Stage Group			<0.001			<0.001
IV	Reference					
III	0.653	0.633 to 0.673	<0.001	0.623	0.603 to 0.644	<0.001
II	0.521	0.465 to 0.583	<0.001	0.477	0.422 to 0.539	<0.001
I	0.307	0.283 to 0.333	<0.001	0.255	0.233 to 0.279	<0.001
Surgery			<0.001			<0.001
No	Reference					
Yes	0.500	0.461 to 0.543	<0.001	0.495	0.453 to 0.540	<0.001
Radiation recode			<0.001			<0.001
No	Reference					
Yes	0.685	0.664 to 0.706	<0.001	0.695	0.673 to 0.718	<0.001
Age at diagnosis	1.013	1.011 to 1.015	<0.001	1.012	1.010 to 1.013	<0.001
Marital status			<0.001			<0.001
Separated	Reference					
Divorced	1.023	0.917 to 1.142	0.681	1.021	0.911 to 1.144	0.721
Single	1.011	0.906 to 1.128	0.839	0.997	0.890 to 1.117	0.956
Windowed	0.980	0.878 to 1.094	0.717	0.984	0.878 to 1.103	0.780
Unknown	0.912	0.807 to 1.030	0.137	0.918	0.808 to 1.041	0.182
Married	0.907	0.815 to 1.010	0.075	0.918	0.822 to 1.026	0.132
Unmarried or domestic partner	0.825	0.564 to 1.205	0.319	0.830	0.560 to 1.230	0.354
High school education			0.008			0.001
<7	Reference					
7-12	1.001	0.943 to 1.062	0.975	1.007	0.947 to 1.071	0.833
13-20	1.001	0.939 to 1.067	0.967	1.015	0.950 to 1.085	0.659
>21	0.942	0.879 to 1.010	0.095	0.941	0.875 to 1.012	0.103
Median household income			<0.001			0.001
>63000	Reference					
48000-62999	1.018	0.985 to 1.053	0.287	1.027	0.992 to 1.064	0.133
38000-47999	1.053	1.009 to 1.100	0.018	1.055	1.009 to 1.104	0.019
<38000	1.124	1.062 to 1.190	<0.001	1.131	1.066 to 1.200	<0.001
Chemotherapy recode			<0.001			<0.001
No	Reference					
Yes	0.435	0.424 to 0.447	<0.001	0.436	0.424 to 0.448	<0.001

Abbreviations: SCLC: Small cell lung cancer; NOS: Not otherwise specified; HR: hazard ratio; CI: Confidence interval.

**Table 3:** Multivariate analysis using a Cox proportional hazards model in SCLC patients >80.

**Variable**	**Multivariate analysis**					
	**OS**			**CSS**		
	**HR**	**95% CI**	***P***	**HR**	**95% CI**	***P***
Sex			<0.001			<0.001
Male	Reference					
Female	0.883	0.831 to 0.939	<0.001	0.896	0.841 to 0.955	<0.001
Primary Site			0.002			0.003
Main bronchus	Reference					
Upper lobe	0.828	0.739 to 0.927	0.001	0.812	0.722 to 0.913	0.001
Middle lobe	0.820	0.683 to 0.985	0.033	0.819	0.677 to 0.991	0.404
Lower lobe	0.872	0.774 to 0.983	0.024	0.858	0.757 to 0.972	0.016
NOS	0.936	0.824 to 1.063	0.308	0.925	0.810 to 1.056	0.248
Overlapping lesion	1.080	0.846 to 1.378	0.536	1.025	0.792 to 1.326	0.852
Laterality			0.001			<0.001
Bilateral, single primary	Reference					
Right-origin of primary	0.912	0.681 to 1.220	0.533	0.926	0.683 to 1.254	0.618
Left-origin of primary	0.987	0.737 to 1.323	0.932	1.009	0.744 to 1.369	0.956
Paired sit	0.716	0.524 to 0.977	0.035	0.704	0.508 to 0.975	0.035
Only one side - side unspecified	0.636	0.380 to 1.064	0.085	0.660	0.388 to 1.122	0.124
Not a paired site	0.917	0.458 to 1.833	0.805	0.890	0.429 to 1.844	0.753
Stage Group			<0.001			<0.001
IV	Reference					
III	0.739	0.689 to 0.792	<0.001	0.717	0.667 to 0.771	<0.001
II	0.539	0.428 to 0.679	<0.001	0.480	0.373 to 0.618	<0.001
I	0.369	0.318 to 0.429	<0.001	0.326	0.277 to 0.384	<0.001
Surgery			<0.001			<0.001
No	Reference					
Yes	0.536	0.406 to 0.709	<0.001	0.596	0.446 to 0.796	<0.001
Radiation recode			<0.001			<0.001
No	Reference					
Yes	0.678	0.631 to 0.728	<0.001	0.680	0.630 to 0.733	<0.001
Median household income			<0.001			0.014
>63000	Reference					
48000–62999	1.050	0.981 to 1.123	0.162	1.047	0.976 to 1.125	0.201
38000–47999	1.159	1.061 to 1.266	0.001	1.157	1.055 to 1.269	0.002
<38000	0.994	0.867 to 1.139	0.929	0.967	0.837 to 1.116	0.643
Chemotherapy recode			<0.001			<0.001
No	Reference					
Yes	0.424	0.397 to 0.451	<0.001	0.415	0.389 to 0.444	<0.001

Abbreviations: SCLC: Small cell lung cancer; NOS: Not otherwise specified; HR: hazard ratio; CI: Confidence interval.

## DISCUSSION

The results of our study demonstrated the benefit of chemotherapy in both young and elderly SCLC patients. Although chemotherapy could benefit OS and LCSS regardless of age, patients older than 80 years tended to reject chemotherapy compared with patients younger than 80 years. Besides, the results of our COX analysis showed that male sex, an increased stage and grade, and no surgery or radiation were associated with worse prognoses. In < 80 years group, Caucasian patients who were diagnosed late or at an older age tended to have worse prognoses.

More studies have paid increasing attention to the relationship between chemotherapy and survival of SCLC patients, especially in elderly SCLC patients. Elegbede et al. reviewed 404 SCLC patients managed at a tertiary cancer center in Canada from 2010 to 2016 and found that chemotherapy benefited survival of SCLC patients, especially those in extensive stages (HR 0.33; 95% CI 0.22–0.48; *P* < 0.01) [19]. Another study developed a nomogram prognostic model based on a large cohort of 24,680 SCLC patients from the National Cancer Database (NCDB), and their result showed that chemotherapy was beneficial to improving prognosis (HR 0.35; 95% CI 0.33–0.36; *P* < 0.001) [20]. A study from the Japanese Joint Committee of Lung Cancer Registry [21] reported that OS of their 228 SCLC patients aged > 75 years who received second-line chemotherapy was 13.9 weeks, which was significantly higher than 7.5 months in those who received supportive care alone in a previous randomized controlled study [22]. A meta-analysis recruiting 14 relevant randomized clinical trials from the Medline and Cochrane databases reported that the 1-year OS improved from 30% to 39% and the 2-year OS improved from 10% to 14% in SCLC patients who received maintenance chemotherapy [23]. Several previous studies concluded that patients of advanced age tended to reject chemotherapy, which is consistent with our study. By reviewing a number of retrospective studies, Deppermann et al. [24] concluded that elderly patients were often offered only suboptimal or no treatment. Researchers from the British Columbia Cancer Agency carried out a retrospective review on 174 patients with SCLC between 1991 and 1999, and categorized them into three age groups: < 65 years (*n* = 55), 65–74 years (*n* = 76), and ≥ 75 years (*n* = 43). The results displayed that elder patients tended to fail to complete an “optimal” course (intravenous regimens, more than 85% total doses, 4+ cycles, and less than 2 weeks total treatment delays) of first-line chemotherapy (*P* < 0.05), and patients >65 years were less likely to administer second-line chemotherapy (*P* < 0.05) [16]. In the light of a recently published retrospective analysis of SCLC patients at IPO-Porto, Portugal's largest oncology hospital, the median age of patients who received platinum doublet chemotherapy was lower than the respective full populations (LS-SCLC: 64 years vs. 70 years; ES-SCLC: 63 years vs. 64 years), suggesting that age may affect the patient’s decision whether to receive chemotherapy [25]. Stacey et al. [11] reported that PS and the presence of comorbidities were the most common factors that affected clinical oncologists not to recommend chemotherapy, and most patients rejected chemotherapy mainly because of their concerns about toxicity. Our COX analysis also demonstrated that surgery and radiation were positively associated with better survival, which is consist with other recent studies [26–29]. In a retrospective analysis of 366 SCLC patients receiving chemotherapy or chemoradiotherapy [28], Kanaji et al. found in LS-SCLC patients with idiopathic pulmonary fibrosis (IPF), chemoradiotherapy related to better progression-free survival (PFS) (281 days vs. 146 days, *P* = 0.0471) and OS (1163 vs. 355 days, *P* = 0.0012) compared with chemotherapy only. A comparative study involving 37 postoperative patients and 37 patients without surgery who both received chemotherapy of “etoposide + cisplatin or carboplatin” showed that the 1, 3, 5-year survival rates were significantly different (1-year survival rate: 72.97% vs. 54.05%; 2-year survival rate: 35.13% vs. 13.51%; 3-year survival rate: 21.62% vs. 5.41%) [29]. Elegbede et al. analyze the survival of 404 SCLC patients and fund that surgery benefited OS of LS-SCLC patients compared with no treatment (40 months vs. 8 months), and chemotherapy combined with thoracic radiotherapy correlated with longer OS compared with chemotherapy alone (13 months vs. 9 months) [19].

Our study investigated a large cohort of 57,460 SCLC patients from real world data. Although many other studies have also addressed the effect of chemotherapy on the survival prognosis of SCLC patients [16, 17, 19, 20, 22], they were mostly based on limited sample sizes, and there have been few investigations on the prognosis of different age subgroups. Given aging of the population, patients older than 80 years will account for a greater proportion. Therefore, the results of our study may provide useful information for clinical oncologists and elderly patients in choosing treatment options. Additionally, we also analyzed the impact of other factors on patient survival, which may also guide patient management. However, several deficiencies in our research need to be mentioned. Despite the large sample size of the SEER, the smoking status and PS are not available in the database, knowing that the former is strongly associated to SCLC [30, 31], and the latter has also proved to be related to the prognosis of SCLC patients [32]. It is possible that patients who smoke or have a low PS score were included in the non-chemotherapy group, which may induce biases in the results. Concurrently, reasons for not choosing chemotherapy, or potential factors affecting the receipt of chemotherapy, such as the nutritional status, cognitive function and geriatric syndrome, are not included in the SEER, either. These limitations need to be refined in future validation using data from hospitals, or randomized controlled trials.

## CONCLUSIONS

To conclude, chemotherapy is a beneficial choice for patients with SCLC over 80 years old, although elderly patients are less likely to receive chemotherapy. Age should not be the main basis for deciding whether to receive chemotherapy or not.
